# Large ossifying fibroma of jaw bone: a rare case report

**DOI:** 10.11604/pamj.2018.30.306.15877

**Published:** 2018-08-31

**Authors:** Vinay Kharsan, Ramnik Singh Madan, Pankaj Rathod, Abhishek Balani, Sumit Tiwari, Saurabh Sharma

**Affiliations:** 1 Department of Oral and Maxillofacial Surgery, New Horizon Dental College and Research Institute, Bilaspur, Chhattisgarh, India

**Keywords:** Ossifying fibroma, fibro-osseous lesion, cemento-fying fibroma

## Abstract

Ossifying fibroma (OF) is classified as, and behaves like, a benign bone neoplasm. It is often considered to be a type of fibro-osseous lesion (FOL). It can affect both mandible and the maxilla, particularly the mandible. This bone tumour consists of highly cellular, fibrous tissue that contains varied amounts of bone or cementum resembling calcified tissue. Present case is an unusual report of central ossifying fibroma involving the left side of mandible in an 18 year old female patient, who presented to the department with a painless hard swelling. The lesion was treated by surgical resection and reconstruction.

## Introduction

Ossifying fibroma (OF), a benign bone neoplasm often considered to be type of fibro-osseous lesion (FOL) can affect both the mandible and maxilla, but is more frequently seen in the mandible with an incidence of 70-90% of the cases [1]. Clinically this tumour appears as a slowly growing intrabony mass which is often asymptomatic and rarely large enough to cause facial asymmetry [2]. It is commonly seen in the third and fourth decades of life. Radiographically, the lesion is often unilocular and well defined with varying degrees of mineralization. This bone tumour consists of highly cellular, fibrous tissue that contains varying amounts of calcified tissue resembling bone, cementum or both [3]. In 1968, Hamner et al analysed and classified 249 cases of fibro-osseous jaw lesions of periodontal membrane origin. In 1973, Waldron and Giansanti reported 65 cases (of which 43 cases had adequate clinical histories and radiographs) and concluded that this group of lesions was best considered as a spectrum of processes arising from cells in the periodontal ligament [4]. In 1985, Eversole *et al* described the radiographic characteristics of central ossifying fibroma, and noted two major radiographic patterns, expansile unilocular radiolucent pattern and multilocular configuration [5]. Treatment comprises of enucleation and curettage or surgical resection for larger lesions. The rate of recurrence is usually low [6]. Although some cases of Ossifying Fibroma have been reported in the literature, massive expansile lesions measuring more than 10cm, like the case in this study is rare. The adequate radiographs of the case presented can further help us to understand the variable radiographic appearances of this lesion.

## Patient and observation

**Figure 1:**
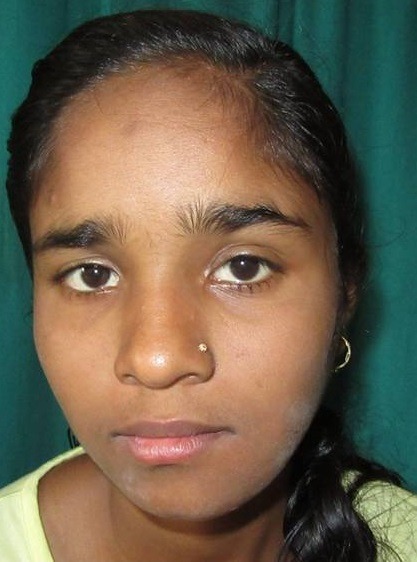
Preoperative extraoral view

**Figure 2:**
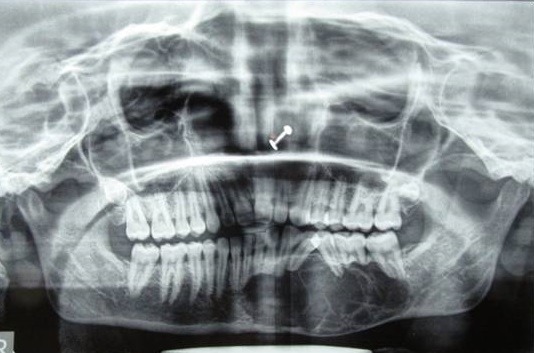
Panoramic view

**Figure 3:**
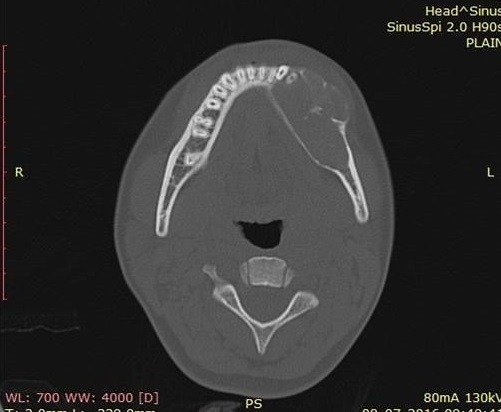
Computed tomography view

**Figure 4:**
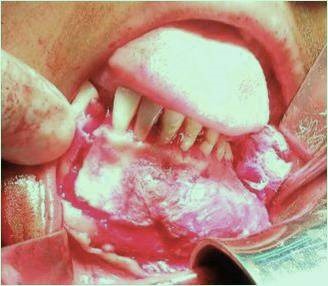
Incision and exposure

**Figure 5:**
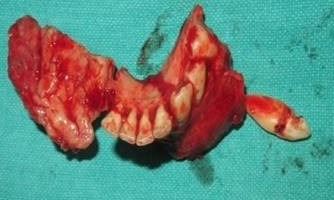
Resected specimen

**Figure 6:**
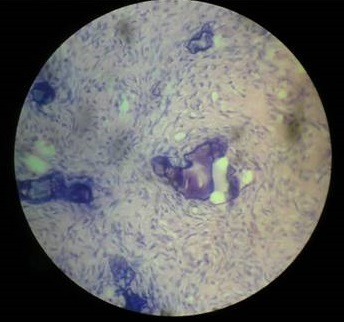
Postoperative panoramic view

A female patient aged 18 years reported to our department with a chief complaint of painless swelling in the lower left side of the face since 5 months, her history revealed that swelling started spontaneously 5 months back and was slowly progressing in size to current status. Extra oral examination revealed solitary hard swelling with diffuse borders in lower left side of the face, approximately 8cm X 7cm in size. The swelling was extending from 1cm anterior to angle of mandible to the symphysis region. Swelling was non-tender and overlying skin was inflamed with temperature slightly raised (Figure 1). Intraorally, bicortical expansion of the swelling with vestibular obliteration was noted from 33 to 36 regions. OPG revealed a well defined multi-locular radiolucency extending from 41 to 37 regions with multiple internal septae and locules. Root resorption with respect to 41, 31, 32, 33, 34, 35, 36 and 37 with lateral displacement of 33, 34, 35 and thinning of lower border of mandible was also noted (Figure 2). Computed tomography showed the buccal and lingual cortical expansion with destruction (Figure 3). Incisional biopsy was done and histopathological lesion showed dense connective tissue stroma with areas of immature bone suggestive of Fibro-Osseous Lesion. After obtaining informed consent from both the patient and her relatives, resection of tumor and reconstruction was planned under GA. An intraoral degloving incision was given from lower right premolar to lower left second molar region; mucoperiosteal flap was raised with proper care to preserve the mental nerve (Figure 4). The lesion was exposed up to the inferior border, vertical stop cut was made distal to 37 to 43 and horizontal cuts were made inferiorly to preserve the lower border. Buccal and lingual cortical plates were osteotomize and the segment was removed in total (Figure 5). Reconstruction plate was modified, adapted and fixed with screws over the site after achieving proper occlusion. Thorough irrigation was done, haemostasis was achieved and closure was done with 3-0 vicryl sutures. Patient was followed up regularly every month for 1 year and post-operative healing was satisfactory (Figure 6).

**Figure 7:**
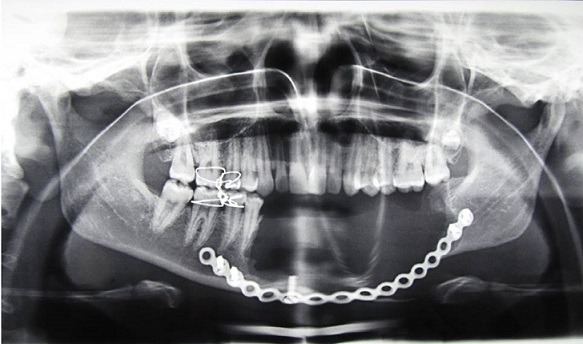
Histopathological view

**Histopathology report:** Microscopic examination of Hematoxylin & Eosin stained section showed highly cellular connective tissue composed of interlacing fascicles of plump and proliferating spindle shaped fibroblast with delicate collagen fibres. Area of multiple and varying sized dystrophic calcifications were evident (Figure 7).

## Discussion

grouped together since 1968 [7]. In 1971, World Health Organization (WHO) classified four types of cementum-containing lesions; fibrous dysplasia, ossifying fibroma, cementifying fibroma and cement- ossifying fibroma [8]. According to the second WHO classification, benign fibro-osseous lesions in the oral and maxillofacial regions were divided into two categories, osteogenic neoplasm and non-neoplastic bone lesions in which cementifying ossifying fibroma belonged to the former category [9]. However, the term "cementifying ossifying fibroma" was reduced to ossifying fibroma in the recent classification in 2005 [10]. The origin of ossifying fibroma is thought to be from the periodontal membrane [11]. Some ossifying fibromas contain prevalent cementum-like calcifications and others show only bony material, but a mixture of the two types of calcification is commonly seen in a single lesion [12, 13]. It can occur at any age, however, many authors confirmed that lesions involving the jaws tend to occur in middle-aged patients [2, 7-9] and it is important to note that, (OF) of the jaw bone has a high predilection for females [14]. Ossifying fibroma predominantly affects the craniofacial bone and rarely involves the long bones. Of the craniofacial bones, mandible is the most commonly involved site, typically inferior to the premolars and molars [15]. Radiographically these tumors present with a number of patterns depending upon the degree of mineralization [15]. Depending on the amount of calcified material produced in the tumor, it may appear as unilocular or multilocular radioopaque image or a radiolucency with mixed density of opacified material. In some cases it is associated with root resorption and displacement of adjacent teeth [15].

Histologically, ossifying fibromas consist of fibrous tissue that exhibits varying degrees of cellularity and contains mineralized mineral. The hard tissue portion may be in the form of trabeculae of osteoid and bone or cementum resembling basophilic and poorly cellular spherules. The bony trabeculae vary in size and frequently demonstrate a mixture of woven and lamellar pattern. Peripheral osteoid and osteoblastic rimming are usually present. The spherules of cementum-like material often demonstrate peripheral brush borders that blend into the adjacent connective tissue. Significant intralesional hemorrhage is unusual [16]. Differential diagnosis of ossifying fibroma is complicated and includes fibrous dysplasia, calcifying odontogenic cyst, cementoblastoma, chondrosarcoma and osteosarcoma. It can be established based upon the clinical and radiographic examination. Radiographically, ossifying fibroma always presents with well defined margins. Fibrous dysplasia has a classical ground glass appearance. Other conditions like calcifying odontogenic cyst or cementoblastomas appear as mixed periapical image associated with vital teeth [16]. Enucleation and curettage is the initial treatment of choice for small ossifying fibromas. Surgical resection is indicated for larger lesions. Prognosis is usually good as rate of recurrence is not very high and a periodic long term follow up is essential [16]. In our case, surgical resection and reconstruction was carried out and follow up revealed satisfactorily normal healing.

## Conclusion

We reported and treated an unusual case of a large Ossifying fibroma in a 18 year old female patient who presented with a multilocular radiolucent swelling in lower left side of the mandible. Based on our experience, we suggest that proper correlation of the clinical, radiological and histological features is necessary to establish a definitive diagnosis, as well as for proper surgical intervention. As reported in literature, the rate of recurrence is not very high but long term periodic follow up is warranted.
